# Human *Rickettsia felis* infections in Mainland China

**DOI:** 10.3389/fcimb.2022.997315

**Published:** 2022-09-23

**Authors:** Zhongqiu Teng, Na Zhao, Ruotong Ren, Xue Zhang, Zhenshan Du, Pengfei Wang, Tian Qin

**Affiliations:** ^1^ The State Key Laboratory of Infectious Disease Prevention and Control, National Institute for Communicable Disease Control and Prevention, Chinese Center for Disease Control and Prevention, Beijing, China; ^2^ Foshan Branch, Institute of Biophysics, Chinese Academy of Sciences, Beijing, China; ^3^ Institute of Innovative Applications, MatriDx Biotechnology Co., Ltd., Hangzhou, China; ^4^ Department of Respiratory Medicine, Shanxi Bethune Hospital, Shanxi Academy of Medical Sciences, Tongji Shanxi Hospital, Third Hospital of Shanxi Medical University, Taiyuan, China

**Keywords:** *Rickettsia felis*, flea-borne spotted fever, China, human infection, fever of unknown origin

## Abstract

We identified four flea-borne spotted fever cases caused by *Rickettsia felis* in a retrospective survey of 182 patients with fever of unknown origin (FUO) in China between 2021 and 2022. The clinical signs and symptoms of the patients were similar to those of other rickettsioses, including fever, rash, and liver and kidney dysfunction. All four patients in the present study developed pneumonia or lung lesions after *R. felis* infection. The cases of *R. felis* infection, a neglected infectious disease, were sporadic in multiple provinces of the country. The high prevalence (2.14%, 4/187) of *R. felis* among patients with FUO highlights the risk posed by this pathogen to public health in China.

## Introduction

*Rickettsia felis*, a causative agent of emerging flea-borne spotted fever, can infect various vectors and hosts (Legendre and Macaluso, 2017). In contrast to other rickettsiae, which have one or two specific vector species, *R. felis* can be carried by several species of fleas, ticks, mites, lice, and mosquitoes (Reif and Macaluso, 2009; Parola et al., 2013). The cat flea, *Ctenocephalides felis*, is believed to be the primary vector and reservoir host; however, the potential role of additional vectors in disease transmission cannot be ignored (Angelakis et al., 2016). In China, the prevalence of *R. felis* in *C. felis* was found to be 95% (Zhang et al., 2014).

**Figure 1:**
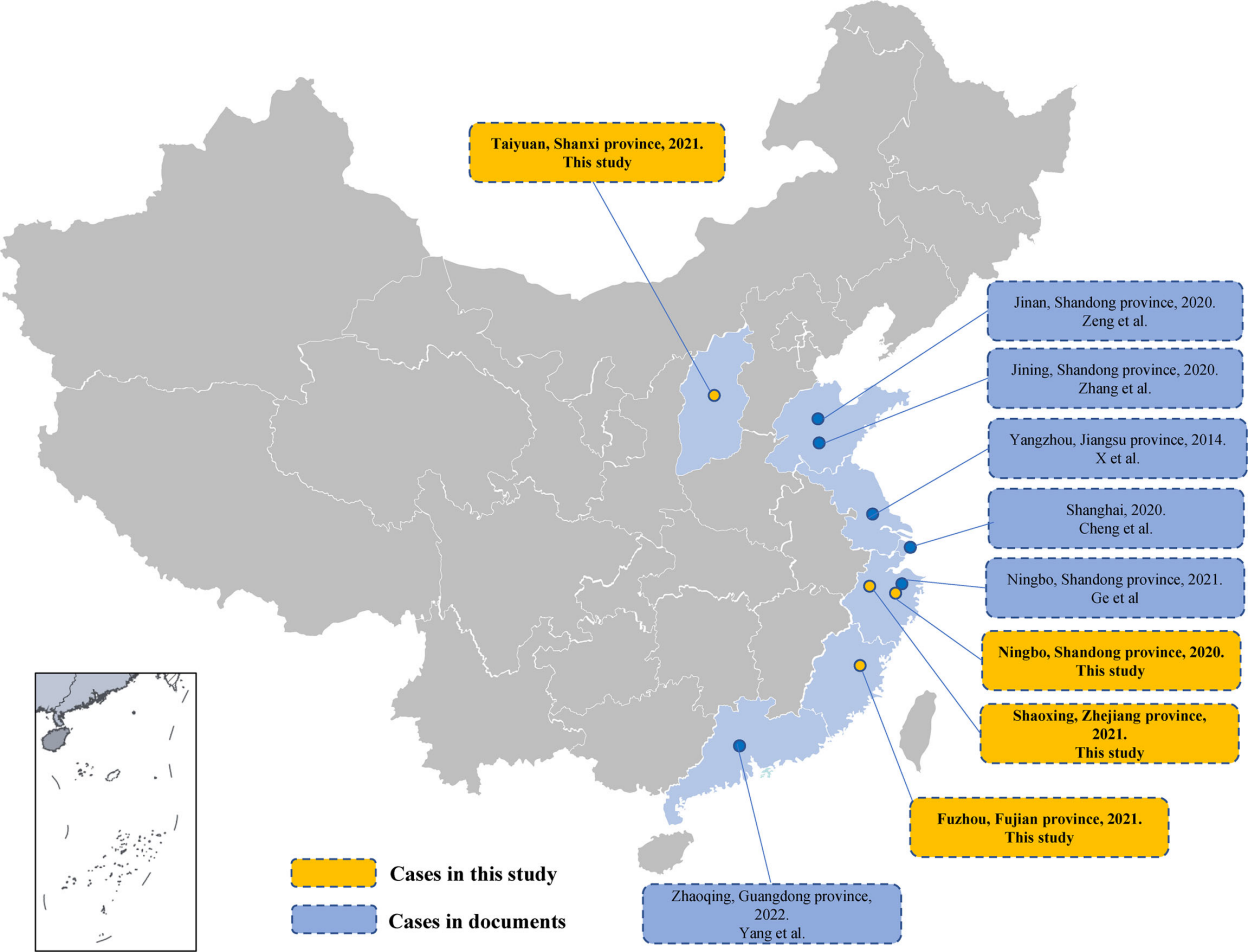
Human infection by *Rickettsia felis* in mainland China. Provinces where human cases of *R. felis* infection have been found are labeled in light blue. Curved lines indicate provincial borders.

After the first human infection in 1994 in the USA, hundreds of flea-borne spotted fever cases have been reported worldwide (Parola et al., 2005; Reif and Macaluso, 2009; Renvoisé et al., 2009; Parola, 2011; Maina et al., 2012). The cases spread across all continents except Antarctica (Brown and Macaluso, 2016; Khan et al., 2020). Recent studies on patients with fever of unknown origin (FUO) in Africa found that up to 15% of blood had detectable levels of *R. felis* (Mediannikov et al., 2013). The first *R. felis* infection case in China was reported in Jiangsu Province *via* PCR analysis in 2014 (Zhang et al., 2014). More cases of rickettsial disease have been diagnosed in recent years owing to the development and application of high-throughput sequencing, and these include five *R. felis* infections in mainland China (Yang X. S. et al., 2021; Ye et al., 2021; Zeng et al., 2021; Zhang et al., 2021; Cheng et al., 2022) (**Figure 1**). These were sporadic cases distributed across several provinces (Shanxi, Zhejiang, and Fujian). However, serological studies have found high *R. felis* infection prevalence (16%) in healthy people (Zhang et al., 2014). Therefore, we should be alert to potential outbreaks of flea-borne spotted fever.

From April 2021 to June 2022, four cases of *R. felis* infection were found in patients with FUO in a retrospective tick-borne disease survey project led by the National Institute for Communicable Disease Control and Prevention (ICDC), and their medical records were reviewed.

## Materials and methods

### Sampling and ethics statements

This study was part of a tick-borne disease surveillance project led by the ICDC. From April 2021 to June 2022, plasma or bronchoalveolar lavage fluid (BALF) of 187 patients with FUO from primary hospitals in six provinces (Hubei, Shanxi, Fujian, Zhejiang, Anhui, and Hunan) were collected for multi-pathogen screening. Experimental protocols for collecting human clinical samples and isolating *Rickettsia* from the samples were approved by the Ethical Review Committee of the ICDC and the Chinese Center for Disease Control and Prevention (China CDC). Written consent was obtained from all participants before the study regarding any potential identifiable images or data included in this article.

### DNA extraction

BALF (1 mL) was first centrifuged at 13,000 × *g* for 10 min. The pellet was resuspended in 200 μL of phosphate-buffered saline (GE HyClone, USA). DNA was extracted using the DNeasy Mini Kit (Qiagen, Germany), following the manufacturer’s instructions. DNA was extracted from 3 mL of collected plasma using the QIAamp Circulating Nucleic Acid Kit (Qiagen, Germany). The quality and quantity of the DNA were evaluated using a NanoDrop spectrophotometer (Thermo Scientific, USA), and the DNA samples were stored at −80°C until use.

### Molecular diagnosis and amplicon sequencing

The DNA samples were screened using a real-time PCR method described previously for zoonotic pathogens, including *Rickettsia*, *Anaplasma*, *Ehrlichia*, and *Coxiella*, with the following reaction conditions: 95°C for 3 min (one cycle); 95°C for 10 s and 68°C for 20 s (40 cycles) (Huang et al, 2021; Jiao et al, 2021; Teng et al, 2021). Positive samples were confirmed, and pathogens were typed by PCR or semi/nested PCR, followed by Sanger sequencing (**Table 1**). Sequences were aligned against those in the GenBank database with the NCBI BLAST tool and phylogenetic trees were generated using the maximum-likelihood method implemented in MEGA 6 software (http://www.megasoftware.net) with 1000 bootstrap replicates.

**Table 1:** Primers used in this study.

Pathogen	Target gene	Primer name	Sequence	Size (bp)	Reference
*Rickettsia* spp.	17-kD	R-r17k1-F	TTTACAAAATTCTAAAAACCAT	539	(Ishikura et al., 2003)
		R-r17k539-R	TCAATTCACAACTTGCCATT		
		R-r17k90-F	GCTCTTGCAACTTCTATGTT	450	
		R-r17k539-R	TCAATTCACAACTTGCCATT		
	*omp*A	R-r190k.71p	TGGCGAATATTTCTCCAAAA	650	(Ishikura et al., 2003)
		R-r190k.720n	TGCATTTGTATTACCTATTGT		
		R-r190k.71p	TGGCGAATATTTCTCCAAAA	532	
		R-r190k.602n	AGTGCAGCATTCGCTCCCCCT		
	*omp*B	Rc.rompB.4362p	GTCAGCGTTACTTCTTCGATGC	475	(Choi et al., 2005)
		Rc.rompB.4,836n	CCGTACTCCATCTTAGCATCAG		
		Rc.rompB.4,496p	CCAATGGCAGGACTTAGCTACT	267	
		Rc.rompB.4,762n	AGGCTGGCTGATACACGGAGTAA		
	*sca*4	R-sca4-1072F	TCGGTTGAACCACCTCAGCATA	711	(Li et al., 2020)
		R-sca4-1782R	TGTGCCGGACTGAGAACTTGGA		
		R-sca4-1146F	GGCTTCACAAATGCCACAGTCG	389	
		R-sca4-1154R	TCTGCTGTTTTTGCTGCGGCTC		
*Rickettsia felis*	*glt*A	Rfelis-gltA-F1	GCAAGTATTGGTGAGGATGTAATC	886	(Hii et al., 2011)
		Rfelis-gltA-R1	CTGCGGCACGTGGGTCATAG		
		Rfelis-gltA-F2	GCGACATCGAGGATATGACAT	654	
		Rfelis-gltA-R2	GGAATATTCTCAGAACTACCG		
	*fel*B*	RfelB-F	TAATTTTAACGGAACAGACGGT	97	(Phomjareet et al., 2020)
		RfelB-R	GCCTAAACTTCCTGTAACATTAAAG		
		RfelB-P	FAM-TGCTGCTGGTGGCGGTGCTA-BHQ		

*Real-time PCR method.

For *R. felis-*positive samples, *R. felis* species-specific real-time PCR methods and nested PCR with primers targeted to *R. felis* species-specific citrate synthase (*glt*A) were performed.

The sequences were deposited in GenBank: 17-kD (accession nos. ON973850 and ON973851), *omp*B (accession nos. ON973854, ON973855, ON973856 and ON973857), and *glt*A (accession nos. ON973852 and ON973853).

### Serological tests

The first serum sample collected when each patient first presented at the hospital was analyzed for IgG against *R. felis* using indirect immunofluorescence assay (IFA) kits, following the instructions of the manufacturer (Focus, Cypress, USA).

## Results

### Multipathogen screening and serological tests

One hundred and eighty-seven clinical samples, including 167 plasma and 20 BALF samples, were collected from patients with FUO. Based on real-time PCR, 18 samples were positive for *Rickettsia*, and no other tick-borne pathogens were detected. All positive samples were tested with nested PCR targeting rickettsial genus-specific genes (17-kD, *omp*A, *omp*B, and *sca*4 genes). The resulting high-fidelity PCR products were Sanger sequenced and aligned against the GenBank database using the NCBI BLAST tool. We finally found 14 *R. japonica*-positive specimens (plasma samples) and four *R. felis*-infected patients (two plasma and two BALF).

To confirm the infection of *R. felis* in the four patients, *R. felis* species-specific real-time PCR was performed, and the *gltA* gene was amplified from the DNA samples taken from these patients by nested PCR using *R. felis* species-specific primers. As shown in **Table 2**, real-time PCR detected *R. felis* in all four patients. Three genes (*omp*B, *glt*A and 17-kD) were amplified from case 1. Two genes were amplified from case 2 (*omp*B and *glt*A) and case 4 (*om*pB and 17-kD). For the sample from case 3, only *omp*B was amplified. BLAST analysis revealed that the amplicon sequences were all 100% similar to the sequences of the homologous genes of *R. felis*.

**Table 2:** Results of real-time PCR and semi/nested PCR.

Case no.	Type of sample	Real-time PCR	*omp*A	*omp*B	*Sca*4	*glt*A	17-kD
1	BALF	+	–	+	–	+	+
2	Plasma	+	–	+	–	+	
3	BALF	+	–	+	–	–	–
4	Plasma	+	–	+	–	–	+

**Table 3:** Clinical characteristics and laboratory test results of the patients with *Rickettsia felis* infection.

Patient	1	2	3	4
Sex	female	Male	female	female
Age	59	58	54	81
Location	Zhejiang Province	Fujian Province	Zhejiang Province	Shanxi Province
Onset date	May	April	May	June
Fever	present	Present	present	present
CRP	increased	Increased	increased	increased
Leukopenia	absent	Present	present	absent
Rash	absent	Present	present	present
Skin lesions	absent	absent	present	present
Liver dysfunction	absent	absent	present	present
Kidney dysfunction	absent	absent	absent	present
Lung lesions	present	present	present	present
Effusion	absent	absent	pleural effusion	pericardial, pleural effusion
Arthropod bitten	NA	present	NA	present

**Figure 2:**
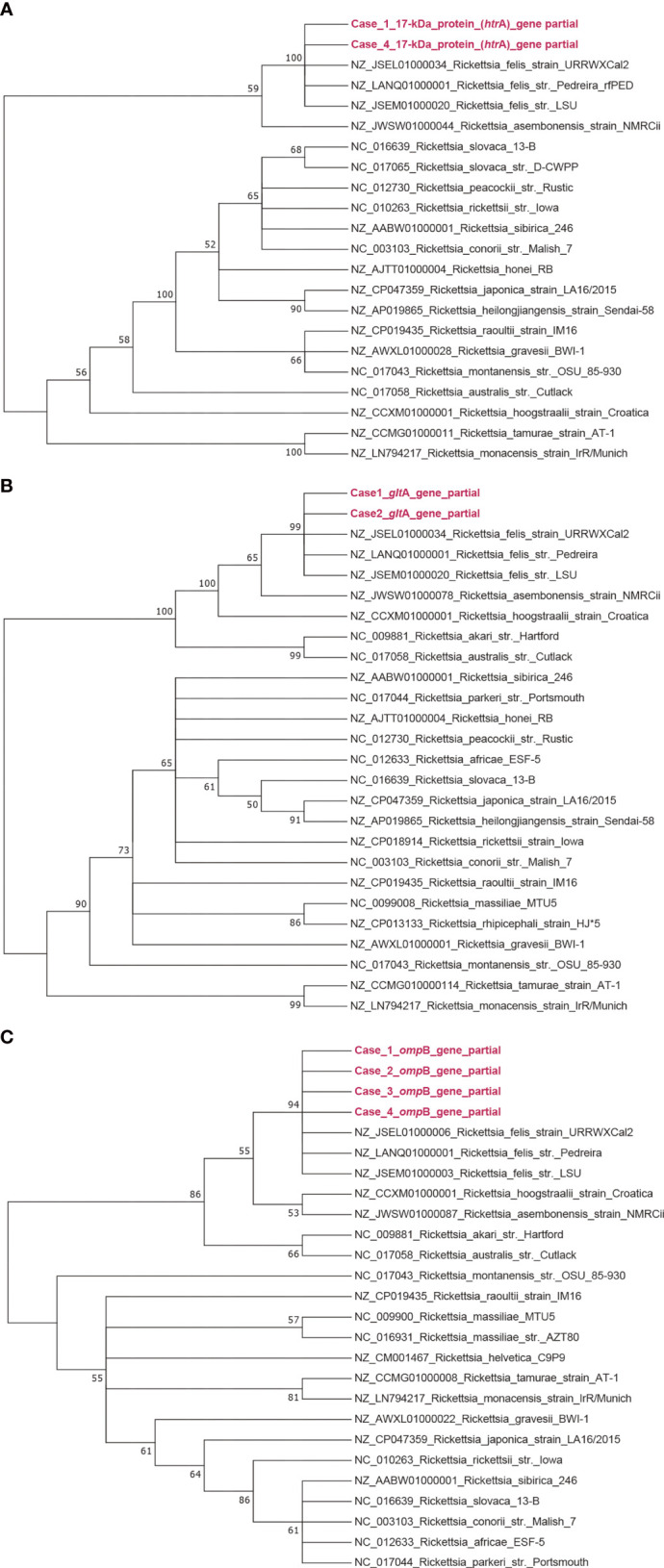
Phylogenetic analysis of *Rickettsia* spp. obtained from this study. Bootstrap consensus phylogenetic tree constructed based on partial sequences of *glt*A **(A)**, *omp*B **(B)**, and 17-kD **(C)** by the neighbor-joining method with 1000 bootstrap replicates.

Phylogenetic trees were generated based on *glt*A, *omp*B, and 17-kD gene sequences in MEGA6 software using the maximum-likelihood method with 1000 replicates for bootstrap testing (**Figure 2**). Phylogenetic analysis showed that the causative agent of spotted fever is most closely related to *R. felis* strain URRWXCal2.

As shown in **Table 4**, two patients (Cases 1 and 4) had a fourfold increase in IgG titer between their acute and convalescent phase samples. However, convalescent serum was not available for the other two patients.

**Table 4:** Serum immunoglobulin antibody titers of the patients with *R. felis* infection.

Case no.	Time of sample collection*		IgG (AP/CP)
	AP	CP	
1	7	14	512/2048
2	7	NA	256/NA
3	10	NA	512/NA
4	8	16	256/1024

AP, acute phase; CP, convalescent phase; NA, not available.

*Days after first symptom onset.

### Case reports

Case 1: A 59-year-old woman presented with a high fever of 38°C for >10 days on admission. Laboratory tests (reference values presented in the Supplementary Text) suggested acute bacterial infection [white blood cells, 7.1×10^9^/L; neutrophils, 82.6%; lymphocytes, 8.6%; C-reactive protein (CRP), 53.3 mg/L; erythrocyte sedimentation rate 65 mm/h] and normal liver and kidney function. However, the patient had an obvious pulmonary infection, multiple exudative foci in both lungs, and micronodular foci in the upper right lung. Levofloxacin, abidol, and loxoprofen sodium tablets were administered to treat dermatomyositis at the initial diagnosis (**Table 3**).

Case 2: A 58-year-old man with mild fever and a body temperature of 37.5°C. There were rashes and hemorrhages throughout his body and no scaling or purpura. The entire body was moist when the patient was admitted to the hospital. Severe leukopenia occurred (white blood cells: 0.67×10^9^/L; neutrophils, 57.6%; lymphocytes, 22.3%). Significant lesions were observed in the lungs. Multiple cystic translucent shadows were seen under bilateral pleura; multiple patchy and small nodular increased density shadows were seen in both lungs with blurred borders, more pronounced in the right lung; multiple lymph node shadows (maximum approximately 1 cm) presented in the mediastinum, bilateral membrane thickening. A CRP level of 120 mg/L and procalcitonin (PCT) of 13.73 ng/mL indicated a serious pathogen infection. Imaging showed that the liver and gallbladder were normal, and there were no signs of ascites.

Case 3: A 54-year-old, female with high fever for >10 days, accompanied by fatigue and dizziness. A skin lesion was found in front of the left lower extremity with slight swelling and scattered red maculopapular rashes on the limbs and trunk. A few days ago, she had a history of unknown arthropod bites. She was first administered azithromycin for anti-infection in primary health care institutions, but the high fever persisted after administration. Imaging revealed bilateral pleural effusion. She had leukopenia (leukocytes 3.410×10^9^/L) and liver function damage [increased aspartate aminotransferase (97 U/L), lactate dehydrogenase (377 U/L), and alanine aminotransferase (92 U/L) levels and decreased albumin (24.8 g/L) and leukocyte ratio (1.18)]. Elevated CRP (30.6 mg/L) and interleukin-6 (19.33 pg/mL) levels suggested an acute-phase infection.

Case 4: An 81-year-old man presented with anamnesis of aortic sclerosis, chronic bronchitis in both lungs, pulmonary hypertension, and multiple coronary calcifications. When visiting a doctor for fever, the highest temperature was 38.5°C. Red rashes were scattered throughout the body. There was a dark red, round induration behind the left ear with pus spots in the middle. The patient recalled being bitten by an arthropod of unknown origin. The white blood cell count of the patient was normal, but neutrophilia (92.4%) occurred. Increased CRP (87.68 mg/L), alanine aminotransferase (40.1 IU/L), aspartate aminotransferase (48.2 IU/L), serum albumin (28.9 g/L), urea (14.9 mmol/L), and serum creatinine (94.9 μmol/L), and pericardial effusion and bilateral pleural effusion suggested that multiple organ dysfunction may have occurred during the infection. The patient also had hypoproteinemia, electrolyte imbalance, hyponatremia, and hypoxemia. The patient was discharged after receiving cefoperazone, sulbactam, ceftriaxone, and supportive treatment.

All the patients had initial symptoms of a sudden fever. A macular rash was observed in three patients (except case 1), and the rash appeared on the limbs and trunk or even developed on the whole body. Two patients (cases 3 and 4) had skin lesions that may have been caused by arthropod bites. Leukopenia was observed in two patients (cases 2 and 3) and neutropenia in two patients (cases 2 and 4). CRP levels were remarkably elevated in all the patients. Liver dysfunction was detected in two patients (cases 3 and 4), including abnormally low plasma albumin levels and abnormally elevated alanine aminotransferase and aspartate aminotransferase levels. Imaging results showed lung lesions in all four patients.

## Discussion

The present study identified four *R. felis* infections in mainland China. The four patients had onset in spring (April to June). *R. feli*s is distributed worldwide, including China, and has multiple vectors and hosts (Brown and Macaluso, 2016). However, reports of flea-borne spotted fever caused by *R. felis* are rare in China (Zhang et al., 2019). Nevertheless, since then, 10 cases have been reported, including four in the present study. Cases of *R. felis* infection were sporadic in multiple regions without an obvious regional distribution. The geographical distribution of the disease may be related to the multiple vectors and broad distribution of pathogens. In addition to four patients diagnosed with flea-borne spotted fever, 14 patients were identified with *R. japonica* exposure in this study, indicating that Japanese spotted fever is also an important rickettsial disease in China.

The clinical signs and symptoms of *R. felis* infection are similar to those of other rickettsioses (tick-borne spotted fever, murine typhus, and scrub typhus), and these include fever, rash, cutaneous eschar, neurological signs, digestive symptoms, cough, and pneumonia (Renvoisé et al., 2009; Maina et al., 2012; Edouard et al., 2014). However, if untreated or improper measures are taken, severe conditions such as hepatomegaly, myocarditis, meningoencephalitis, cerebral edema, and acute and respiratory distress syndrome may cause poor prognosis or even death (Mawuntu et al., 2020; Yang W. H. et al., 2021). In our study, all patients were febrile, but additional symptoms were nonspecific. Three of the four patients had a rash. Pneumonia or lung lesions occurred in all four patients, suggesting that possible rickettsial infections should be considered in bacteria-infected patients with pneumonia of unknown cause. In addition, liver and kidney damage caused by *R. felis* infection should be considered.

Although >100 cases of *R. felis* infection have been diagnosed in many countries in the Americas, Asia, Africa, the Pacific, and Europe since the first detection of *R. felis* in human blood in 1994, the disease-causing transmission mechanism from arthropods to humans remains unclear. In addition to the primary vector, the cat flea, *C. felis*, ticks, mites, and more recently, mosquitoes carrying *R. felis* have been identified, suggesting a complex route of transmission of this *Rickettsia*. Most reported patients were in contact with animals, especially dogs and cats; however, no direct flea contact history was documented (Lim et al., 2012). In the present study, two of the four patients aware of an unknown pest attack had skin lesions, indicating a high risk of animal contact. Mosquitoes may also transmit *R. felis* (Dieme et al., 2015). Correlation research between malaria and *R. felis* infection revealed the potential role of *Anopheles* in the transmission of *R. felis* (Mediannikov et al., 2013; Angelakis et al., 2016). In China, 0.6%–6.0% of mosquito species carry *R. felis*. It is important to consider whether mosquitoes may cause outbreaks of *R. felis* infections (Parola et al., 2016). Almost half of the population of the world is at risk of mosquito-borne diseases and mosquito control is becoming more difficult as the climate changes. We should pay more attention to *R. felis* in mosquitoes in further research.

The present survey has certain limitations. Although four patients were confirmed to be infected with *R. felis* by real-time and semi-nested PCR, nucleic acid fragments of pathogens in some rickettsial-infected patients could not be detected because of low bacterial load. Double serum samples for seroprevalence studies were unavailable for some of the patients.

In summary, we report four cases of *R. felis* infection in China from 2021 to 2022. Reported cases of this neglected infectious disease have increased in recent years. This is a warning to public health workers and physicians who must be aware of infectious diseases, especially for FUO patients with a history of animal contact. It is necessary to perform long-term surveillance and investigation of the local hosts and vectors of *R. felis*, and assess the potential outbreak risks of flea-borne spotted fever.

## Data availability statement

The datasets presented in this study can be found in online repositories. The names of the repository/repositories and accession number(s) can be found in the article/Supplementary Material.

## Ethics statement

The studies involving human participants were reviewed and approved by Ethical Review Committee of the ICDC (ICDC-2019012). The patients/participants provided their written informed consent to participate in this study. Written informed consent was obtained from the individual(s) for the publication of any potentially identifiable images or data included in this article.

## Author contributions

TQ and ZT designed the research. RR, ZD, and PW prepared and provided samples. RR and XZ participated in sample testing. ZT and NZ analyzed the data and wrote the manuscript. All authors read and approved the final manuscript.

## Funding

This work was supported by grants from the National Natural Science Foundation of China (grant No. 81671985), the Science Foundation for the State Key Laboratory for Infectious Disease Prevention and Control of China (grant nos. 2022SKLID209 and 2019SKLID403), and the Public Health Service Capability Improvement Project of the National Health Commission of the People’s Republic of China (grant no. 2100409034).

## Acknowledgments

We thank all the medical institutions, patients, and their families for providing samples.

## Conflict of interest

The authors RR and ZD are employed by MatriDx Biotechnology Co., Ltd.

The remaining authors declare that the research was conducted in the absence of any commercial or financial relationships that could be construed as a potential conflict of interest.

## Publisher’s note

All claims expressed in this article are solely those of the authors and do not necessarily represent those of their affiliated organizations, or those of the publisher, the editors and the reviewers. Any product that may be evaluated in this article, or claim that may be made by its manufacturer, is not guaranteed or endorsed by the publisher.
